# Performance Evaluation of the SAMBA II SARS-CoV-2 Test for Point-of-Care Detection of SARS-CoV-2

**DOI:** 10.1128/JCM.01262-20

**Published:** 2020-12-17

**Authors:** Sonny M. Assennato, Allyson V. Ritchie, Cesar Nadala, Neha Goel, Cuijuan Tie, Lourdes M. Nadala, Hongyi Zhang, Rawlings Datir, Ravindra K. Gupta, Martin D. Curran, Helen H. Lee

**Affiliations:** a Diagnostics for the Real World EU, Ltd., Chesterford Research Park, Great Chesterford, United Kingdom; b Diagnostics for the Real World, Ltd., San Jose, California, USA; c Clinical Microbiology and Public Health Laboratory, PHE Cambridge, Addenbrooke’s Hospital, Cambridge, United Kingdom; d Division of Infection and Immunity, University College London, London, United Kingdom; e Department of Medicine, University of Cambridge, Cambridge, United Kingdom; f Africa Health Research Institute, Durban, South Africa; UNC School of Medicine

**Keywords:** SAMBA II, SARS-CoV-2, point of care, COVID-19

## Abstract

Nucleic acid amplification for the detection of severe acute respiratory syndrome coronavirus 2 (SARS-CoV-2) RNA in respiratory samples is the standard method for diagnosis. The majority of this testing is centralized and therefore has turnaround times of several days. Point-of-care (POC) testing with rapid turnaround times would allow more effective triage in settings where patient management and infection control decisions need to be made rapidly. The inclusivity and specificity of the Simple AMplification-Based Assay (SAMBA) II SARS-CoV-2 test were determined by both *in silico* analyses of the primers and probes and wet testing. The SAMBA II SARS-CoV-2 test was evaluated for performance characteristics. Clinical performance was evaluated in residual combined throat/nose swabs and compared to that of the Public Health England real-time PCR assay targeting the RdRp gene. The SAMBA II SARS-CoV-2 test has an analytical sensitivity of 250 copies/ml for detecting two regions of the genome (open reading frame 1ab [ORF1ab] and nucleocapsid protein [N]). The clinical performance was evaluated in 172 residual combined nose/throat swabs provided by the Clinical Microbiology and Public Health Laboratory, Addenbrooke’s Hospital, Cambridge (CMPHL), which showed an estimated positive percent agreement of 98.9% (95% confidence interval [CI], 93.83 to 99.97) and negative percent agreement of 96.4% (95% CI, 89.92 to 99.26) compared to testing by the CMPHL. The data show that the SAMBA II SARS-CoV-2 test performs equivalently to the centralized testing methods, but with a shorter turnaround time of 86 to 101 min. Point-of-care tests such as SAMBA should enable rapid patient management and effective implementation of infection control measures.

## Abstract (precis)

Nucleic acid amplification for the detection of severe acute respiratory syndrome coronavirus 2 (SARS-CoV-2) RNA in respiratory samples is the standard method for diagnosis. The majority of this testing is centralized and therefore has turnaround times of several days. Point-of-care (POC) testing with rapid turnaround times would allow more effective triage in settings where patient management and infection control decisions need to be made rapidly. The inclusivity and specificity of the Simple AMplification-Based Assay (SAMBA) II SARS-CoV-2 test were determined by both *in silico* analyses of the primers and probes and wet testing.

## INTRODUCTION

Severe acute respiratory syndrome coronavirus 2 (SARS-CoV-2) was first reported in Wuhan, China, in early December 2019 and is the causative agent of coronavirus disease (COVID-19) (1). As of 21 September 2020, it has since spread to over 188 countries/regions around the world (2), causing 961,435 deaths as of 21 September 2020 (https://www.worldometers.info/coronavirus/). It was declared a pandemic by the World Health Organization on the 11 March 2020 (https://www.who.int/director-general/speeches/detail/who-director-general-s-opening-remarks-at-the-media-briefing-on-covid-19---11-march-2020). In Europe, the country with the highest number of deaths is United Kingdom, which as of 21 September 2020 has had 394,257 lab-confirmed cases and 41,777 deaths in all settings (https://coronavirus.data.gov.uk/).

Nucleic acid testing is essential for early diagnosis of SARS-CoV-2 infection, as antibody response is often not detected until approximately 7 to 10 days after onset of symptoms (3). Upper respiratory tract (URT) specimens such as nose and throat swabs generally have high SARS-CoV-2 viral loads upon symptom onset (4). The standard diagnostic test for SARS-CoV-2 in the United Kingdom is done by real-time reverse transcription-PCR (RT-PCR) of the RdRp gene (5) from a combined throat and nose swab sample. The United Kingdom dramatically scaled up testing from 5,000 tests per day in March 2020 to 100,000 tests per day by the end of April. Although this test has good accuracy, the samples must be transported to centralized testing laboratories and batched for processing, which leads to turnaround times of around 48 h or more. This means that treatment and management of severely ill patients may be suboptimal when other causative pathogens are in the differential diagnosis and those requiring admission or triage with possible COVID-19 may be unnecessarily isolated or inappropriately cohorted in a COVID-19 ward. This causes obvious bottlenecks in sample processing; at present, hospitals and regional laboratories are at full capacity, and rapid point-of-care (POC) testing is required.

The Simple AMplification-Based Assay (SAMBA) II nucleic acid testing system was originally designed for HIV testing in POC and resource-limited settings, with CE-marked products for early infant diagnosis (6, 7) and viral load monitoring (8–10). Since 2017, SAMBA HIV tests have been implemented in Uganda, Malawi, Zimbabwe, and the Central African Republic. The SAMBA II SARS-CoV-2 test system has now been developed to specifically detect the presence of the novel coronavirus SARS-CoV-2 in nose and throat swab samples run on the SAMBA II instrument. Test results are available in approximately 1.5 h.

We have here assessed the analytical and clinical performance of the SAMBA II SARS-CoV-2 test using panels and clinical samples.

## MATERIALS AND METHODS

### SAMBA II SARS-CoV-2 test kit and SAMBA II platform.

**FIG 1:**
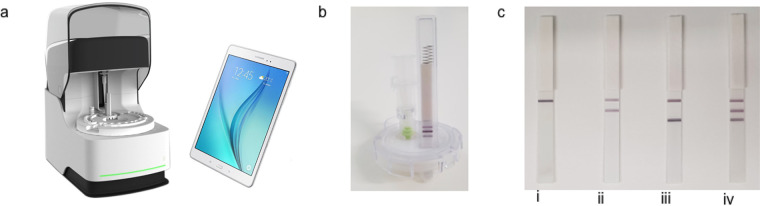
SAMBA II system and possible results. (a) SAMBA assay module and tablet module. (b) SAMBA cartridge showing positive result. (c) Example of SAMBA results: (i) negative, (ii) positive (ORF), (iii) positive (N), and (iv) positive (ORF and N).

The SAMBA II platform is composed of an assay module and a tablet module (Fig. 1a). Each tablet can operate up to 4 assay modules. The SAMBA II SARS-CoV-2 test kit is CE *in vitro* diagnostic (IVD)-marked and contains all of the reagents and disposables required for sample preparation, amplification, and detection preloaded in the cartridges with test times of 86 to 101 min, with strong positive samples stopping at 86 min. The aqueously based SAMBA sample preparation chemistry circumvents the need for alcohol or chaotropic salts. A bespoke freeze-dried synthetic RNA internal control is included in the cartridge and is added during lysis. The target nucleic acid and internal control are amplified from the purified nucleic acid by an isothermal method (NASBA). The SAMBA II SARS-CoV-2 test specifically amplifies two regions of the SARS-CoV-2 genome in open reading frame 1ab (ORF1ab) and the nucleocapsid protein (N). SAMBA detection chemistry allows the visual detection of these two specific amplicons by two lines on a test strip contained within the cartridge (Fig. 1b). A third line detects the internal control to control for false negatives caused by instrument/reagent problems or inhibition. The presence of either test line indicates a positive result (Fig. 1c) in the presence or absence of internal control. The presence of just the internal control indicates a negative result (Fig. 1c). The signal on the test strip is read and interpreted by an integrated camera in SAMBA II and the result reported on the tablet. External positive and negative controls are not provided with the kit, but commercial controls can be used on a daily/weekly basis by sites depending on their own requirements.

### Specimen collection and handling for SAMBA.

The SAMBA II SARS-CoV-2 test kit contains all components required to run the test except swabs. The kit has been validated with combined nasal and throat swab samples using one swab to first collect from the throat then nose. Combined nose and throat swab samples are preferably resuspended in 2 ml of SAMBA SARS-CoV collection (SCoV) buffer, which is provided with the kit. SAMBA SCoV buffer contains detergent and is at a low pH; it has been shown to inactivate SARS-CoV-2 when examined using a pseudovirus luciferase assay as previously described (11) and shows a 4.8-log_10_ reduction in titer when tested with tissue culture fluid containing SARS-CoV-2 (12). However, the system can be used with Remel M4RT or MWE Virocult viral transport medium (VTM) if the sample is diluted 1:2 with SCoV buffer prior to processing. It is recommended that the sample be incubated at room temperature for 10 min to inactivate the sample before loading it into the machine. During this time the cartridges can be loaded and the patient information entered into the tablet. The input volume for the SAMBA test is 300 μl, of which 250 μl is used by the SAMBA II machine as input into the sample preparation.

### *In silico* inclusivity analysis.

The SAMBA-SARS-CoV-2 primers and probes for ORF1ab and N regions were individually evaluated using *in silico* analysis with respect to 157 SARS-CoV-2 sequences in the NCBI database using BLASTN.

### *In silico* specificity analysis.

*In silico* analysis for possible cross-reactions with related human coronaviruses (human coronavirus [hCOV] 229E, human coronavirus OC43, human coronavirus HKU1, human coronavirus NL63, SARS coronavirus, and Middle East respiratory syndrome [MERS] coronavirus) was conducted by mapping primers in the SAMBA II SARS-CoV-2 test individually to the sequences downloaded from the NCBI database.

An *in silico* analysis for possible cross-reactions with other high-priority organisms as indicated in the FDA emergency use authorization (EUA) guidance documents for molecular diagnostic tests (13) was conducted by carrying out a BLASTN search for each of the SAMBA primers and probes against the NCBI databases and retrieving all sequences with homologies of >80%.

### Panels and samples.

Panel members for determination of the limit of detection (LOD) were prepared by making serial dilutions of SARS-CoV-2 RNA from strain 2019-nCoV/Italy-INMI1 from EVAg (code number 008N-03894) in pooled negative combined nose and throat swab samples to target concentrations of 750, 500, 250, 200, 150, and 100 copies/ml.

A coronavirus RNA specificity panel containing hCoV-NL63, hCoV-229E, hCoV-OC43, and MERS-CoV were sourced from the European Virus Archive (EVAg; code number 011N-03868). RNA samples from this panel were tested at >10,000 copies/test in the SAMBA II test in the presence or absence of SARS-CoV-2 RNA at 3× LOD (750 copies/ml) to determine specificity against other human coronaviruses.

Other high-priority organisms indicated by the FDA EUA guidance document for manufacturers (13) were sourced from the Biodefense and Emerging Infections Research Resources Repository (BEI Resources) and the American Type Culture Collection (ATCC) and tested in the presence and absence of SARS-CoV-2 RNA at 3× LOD (750 copies/ml) to evaluate the SAMBA II test for specificity and cross-reaction.

### Contrived clinical samples.

Combined nose and throat swabs were collected from 35 presumed negative individuals using FLOQSwabs (Copan, Italy) and SAMBA SCoV buffer. Thirty contrived positive clinical samples were prepared by spiking known concentrations of SARS-CoV-2 RNA strain 2019-nCoV/Italy-INMI1 (EVAg, Italy) into individual negative specimens to produce final concentrations of 1× LoD (*n* = 3), 2× LoD (*n* = 17), 3× LoD (*n* = 5), 5× LoD (*n* = 3), and 100× LoD (*n* = 2), as recommended in the FDA EUA guidelines (13).

### Clinical evaluation.

The clinical performance of the SAMBA II SARS-CoV-2 test was further evaluated retrospectively in a blind manner with 172 frozen residual combined nose and throat swab samples from the CMPHL. The samples included 88 positives and 84 negatives as initially determined by the CMPHL reference test. These residual samples were from symptomatic individuals with suspected COVID-19 from around the East of England region sent for routine laboratory diagnosis and provided as VTM diluted 1:2 with SAMBA SCoV buffer. In total, 172 samples were tested by the SAMBA II SARS-CoV-2 test, and results were compared to those from the Cambridge RdRp gene (Wuhan) assay on the Rotor-Gene Q real-time PCR instrument routinely used by CMPHL based on the publication by Sridhar et al. (14) but modified by switching the enzyme mastermix used to TaqPath 1-step reverse transcription-quantitative PCR (RT-qPCR) mastermix (catalog no. A15300; Life Technologies). Results are expressed as positive or negative with a threshold cycle (*C_T_*) cutoff of 36 for positive results. Samples were also tested with the PHE Colindale (reference laboratory) assay, essentially as detailed in the publication by Corman et al. (5), which amplifies a different region of the RdRp gene, but performed on the Rotor-Gene Q real-time instrument. In the case of discrepant analysis, the E gene assay described by Corman et al. (5) was also employed to help resolve the status of positive/negative results. The E forward (ACAGGTACGTTAATAGTTAATAGCGT; 400 nM) and E reverse (ATATTGCAGCAGTACGCACACA; 400 nM) primers were identical but the probe, ACACTAGCCATCCTTACTGCG (120 nM), was shortened and a VIC label attached to the 5′ end and an MGB label to the 3′ end. The positive samples had *C_T_* values ranging from 12 to 35.09, with 93% of the samples having *C_T_* values greater than 15, 35% having *C_T_* values greater than 25, 10% greater than 30.

### Research ethics.

Surplus samples obtained from patients known to be symptomatic for COVID-19 and submitted to the CMPHL for routine testing were retrieved before being discarded. These samples were rendered anonymous and provided in a blind manner for the purpose of test validation. Public Health England and NHS Research Ethics Committee have permitted the use of residual samples in this manner, strictly for the purpose of diagnostic assay validation (15). The evaluation was carried out in accordance with the Human Tissue Act (16).

## RESULTS

### Limit of detection.

The limit of detection (LOD) of the SAMBA II SARS-CoV-2 test was determined using serial dilutions of SARS-CoV-2 RNA in pooled negative combined nose and throat swab samples. The initial LOD was determined by testing 6 levels at target concentrations of 750, 500, 250, 200, 150, and 100 copies/ml. Each panel member was tested in replicates of 3 (Table 1). The final LOD was confirmed by testing 250 copies/ml in replicates of 20, of which all were detected. Therefore, the claimed LOD of the SAMBA II SARS-CoV-2 test is 250 copies/ml.

**TABLE 1:** Specificity analysis of SAMBA II SARS-CoV-2 test

Microorganism	Source	Catalog/accession no.	Concn tested
Human coronavirus 229E	Coronavirus RNA specificity panel from EVAg	EVAg Ref SKU: 011N-03868	>10^5^ copies/ml
Human coronavirus OC43	Coronavirus RNA specificity panel from EVAg	EVAg Ref SKU: 011N-03868	>10^5^ copies/ml
Human coronavirus HKNL63	Coronavirus RNA specificity panel from EVAg	EVAg Ref SKU: 011N-03868	>10^5^ copies/ml
SARS-CoV HKU339849	Coronavirus RNA specificity panel from EVAg	EVAg Ref SKU: 011N-03868	>10^5^ copies/ml
MERS coronavirus	Coronavirus RNA specificity panel from EVAg	EVAg Ref SKU: 011N-03868	>10^5^ copies/ml
SARS coronavirus, gamma irradiated and sucrose purified	BEI	NR-9323	1 × 10^5^ PFU/ml
Adenovirus 21	BEI	NR-51436	2.5 × 10^5^ TCID_50_/ml^*a*^
Human metapneumovirus (hMPV), TN/83-1211	BEI	NR-22227	2.8 × 10^5^ TCID_50_/ml
Human parainfluenza virus 1, HPIV1/FRA/27344044/2007	BEI	NR-48681	1.6 × 10^5^ TCID_50_/ml
Human parainfluenza virus 2, Greer	BEI	NR-3229	1.0 × 10^5^ TCID_50_/ml
Bovine parainfluenza virus 3, SF-4	BEI	NR-3234	3.2 × 10^2^ TCID_50_/ml
Human parainfluenza virus 4a, M-25	BEI	NR-3237	1.0 × 10^3^ TCID_50_/ml
Influenza A virus, A/Puerto Rico/8-MC/1934 (H1N1)	BEI	NR-29022	2.8 × 10^5^ TCID_50_/ml
Influenza B virus, B/Sydney/507/2006 (Yamagata lineage)	BEI	NR-32526	8.9 × 10^5^ TCID_50_/ml
Enterovirus 71, Tainan/4643/1998	BEI	NR-471	1.6 × 10^5^ TCID_50_/ml
Human respiratory syncytial virus, A1998/3-2	BEI	NR-28529	1.6 × 10^5^ TCID_50_/ml
Human respiratory syncytial virus, A2001/2-20	BEI	NR-28525	2.8 × 10^4^ TCID_50_/ml
Rhinovirus 20, 15-CV19	BEI	NR-51439	5 × 10^4^ TCID_50_/ml
Chlamydia pneumoniae strain TW-183	ATCC	VR-2282	∼10^6^ cells/ml
Haemophilus influenzae	ATCC	49766	∼10^6^ cells/ml
Legionella pneumophila	ATCC	33152	∼10^6^ cells/ml
Mycobacterium tuberculosis	BEI	NR-49100	∼10^6^ cells/ml
Streptococcus pneumoniae	BEI	NR-51859	∼10^6^ cells/ml
Streptococcus pyogenes	BEI	NR-15271	∼10^6^ cells/ml
Bordetella pertussis	BEI	NR-42460	∼10^6^ cells/ml
Mycoplasma pulmonis	BEI	NR-3858	∼10^6^ cells/ml
Pneumocystis jirovecii (formerly Pneumocystis carinii)	ATCC	PRA-159	∼10^6^ cells/ml
Candida albicans	BEI	NR-29340	∼10^6^ cells/ml
Pseudomonas aeruginosa	BEI	NR-51541	∼10^6^ cells/ml
Staphylococcus epidermidis	ATCC	14990	∼10^6^ cells/ml
Staphylococcus salivarius	BEI	HM-121	∼10^6^ cells/ml

a TCID_50_, 50% tissue culture infective dose.

### Inclusivity.

The SAMBA II SARS-CoV-2 test primers and probes for target 1 (ORF1ab) had a 100% match to all but one available SARS-CoV-2 sequence for this region in the NCBI database (*n* = 157). For this one sequence, a single-nucleotide mismatch was found that maps to the capture probe, with no predicted impact on the assay performance. The primers and probes for target 2 (N) had 100% identity to all available SARS-CoV-2 sequences for this region in the NCBI database (*n* = 157).

### Specificity analysis.

*In silico* analysis for possible cross-reactions with related human coronaviruses (human coronavirus 229E, human coronavirus OC43, human coronavirus HKU1, human coronavirus NL63, SARS coronavirus, and MERS coronavirus) concluded that none of the SAMBA primers had >80% homology to the organisms listed. In addition to *in silico* analysis, the SAMBA II SARS-CoV-2 test was evaluated for specificity by using >10,000 copies/test of hCoV-NL63, hCoV-229E, hCoV HKU339849, hCoV-OC43, MERS-CoV, and SARS-CoV RNA in the presence or absence of 3× LOD of SARS-CoV-2 RNA (750 copies/ml). The SAMBA II SARS-CoV-2 test accurately detected SARS-CoV-2 in spiked samples (at 3× LOD), and all unspiked samples gave negative results, indicating no interference from these microorganisms (Table 1).

An *in silico* analysis for possible cross-reactions with other high-priority organisms listed in the FDA EUA guidance document (13) showed that only one SAMBA probe (N region) had greater than 80% homology (81%) to one of the high-priority organisms (Pneumocystis jirovecii). Wet testing results of these organisms listed by FDA at ∼100,000 genome equivalents (GE)/ml in spiked samples (at 3× LOD) and all unspiked samples, including *P. jirovecii*, showed no cross-reactivity or interference (Table 1).

### Contrived clinical specimens.

Negative (*n* = 35) swab samples from combined nose and throat swab samples collected from 35 individuals were tested with the SAMBA II SARS-CoV-2 test. Spiked samples (*n* = 30) were used to produce contrived positive clinical samples by spiking 30 negative samples with SARS-CoV-2 RNA at 1× LOD (*n* = 3), 2× LOD (*n* = 17), 3× LOD (*n* = 5), 5× LOD (*n* = 3), and 100× LOD (*n* = 2). All 35 negative samples were negative and all 30 spiked positive samples were positive when tested with the SAMBA II SARS-CoV-2 test.

### Clinical evaluation.

The clinical performance of the SAMBA II SARS-CoV-2 test was further evaluated with 172 combined nose and throat samples from symptomatic individuals provided by CMPHL in a blind manner. After initial testing, there were 87 concordant positives, 81 concordant negatives, and 4 discrepant results (3 SAMBA positive and one SAMBA negative) compared with the PHE reference laboratory test (Table 2). Therefore, the positive and negative percent agreements of SAMBA and the PHE reference test were 98.9% (95% confidence interval [CI], 93.83 to 99.97%) and 96.4% (95% CI, 89.92 to 99.26%), respectively (Table 2). The three SAMBA-positive samples were repeat-positive by SAMBA, and on retest by CMPHL they were found to be borderline positive with high *C_T_* values for at least one of the target genes on the Colindale and/or Cambridge (Wuhan) test (Table 3). The one SAMBA-negative sample was negative on repeat by SAMBA but was positive by PHE for RdRp using both the Cambridge (Wuhan) and Colindale assays, with *C_T_* values of 28.87 and 31.18, respectively (Table 3). Therefore, there was just one discrepant sample after retest, a false-negative result for SAMBA. The one SAMBA false-negative result gave a high *C_T_* value on the PHE test (>31), suggesting low viral load; it had been frozen and was diluted (1:2) for SAMBA testing, which may explain the false-negative result.

**TABLE 2:** Clinical performance in 172 clinical samples compared to that of PHE reference test (initial results)

SAMBA SARS-CoV-2 test	PHE reference test		
	+	−	Total
+	87	3	90
−	1	81	82
Total	88	84	172

**TABLE 3:** Discrepant analysis of 4 samples

Sample identifier	SAMBA^*b*^		PHE Colindale assay RdRp Initial (*C_T_*)	PHE Cambridge (Wuhan) assay (UTM sample)			PHE Cambridge (Wuhan) assay (SCoV sample)			Final result
	Initial result	Repeat result		Initial RdRp (*C_T_*)	Repeat RdRp (*C_T_*)	E (*C_T_*)	Initial RdRp (*C_T_*)	Repeat RdRp (*C_T_*)	E (*C_T_*)	
1-25	NEG	NEG	POS (31.18)	POS (28.87)	POS (28.96)	Not tested	Not tested	Not tested	Not tested	POS
2-17	POS	POS	NEG	NEG	NEG	POS (34.99)	POS (33.98)	NEG	NEG	POS
2-25	POS	POS	NEG (36.58)^*a*^	POS (33.62)	NEG	NEG	NEG	NEG	NEG	POS
2-38	POS	POS	NEG	NEG	POS (35.09)	POS (34.06)	NEG	NEG	NEG	POS

a This specimen was assessed as negative by the Colindale RdRp assay; however, when the assay run’s raw data were reexamined and the normal assay control parameters were relaxed (i.e., threshold and endpoint fluorescence) it was weakly positive (shallow curve), with a threshold cycle (*C_T_*) value of 36.58.

b POS, positive; NEG, negative.

## DISCUSSION

The SAMBA II system is a simple “sample in, result out” platform designed for resource-limited and POC settings, and it has been used for HIV testing at the POC (6–10). The instruments can operate in high heat and humidity (10 to 38°C; 5 to 95% humidity), and reagents are stable at room temperature (up to 37°C) for up to 6 months. SAMBA II is simple to operate with just 1 h of training. SAMBA II can be configured to connect to hospital laboratory information management systems (LIMS). The instrument has a small footprint and is controlled by a tablet via Bluetooth anywhere within 10 m of the tablet. Up to 100,000 patient data records can be stored and transferred wirelessly via SMS, 2G to 4G networks, or wifi. Each SAMBA II assay module can process up to 14 samples per day and is therefore ideally suited for POC testing and is not designed for high-throughput screening.

POC molecular tests for SARS-CoV-2 such as SAMBA II are required to quickly triage patients, as centralized testing can take 2 to 5 days for results (17, 18). In addition, POC tests would be extremely useful for nonlaboratory residential settings such as prisons, immigration centers, nursing homes, and rehabilitative centers. The people living in such facilities tend to be vulnerable populations who are at a higher risk for adverse outcome and for infection due to living in close proximity to others (19), and early identification and implementation of increased infection control measures would reduce spread among residents and staff. POC tests would also be extremely useful in other situations where rapid and accurate results are required, e.g., organ donation, hospital admissions, and emergency surgery. POC molecular tests with rapid turnaround, such as SAMBA II, will be essential not only during high rates of infection but also as the country begins to end the lockdown period and localized outbreaks need to be managed quickly and efficiently.

Our data show that the SAMBA II SARS-CoV-2 test is equivalent to centralized testing with excellent positive and negative percent agreements. Samples are inactivated in the SAMBA collection buffer, and results are available within 86 to 101 min at the POC. High sensitivity and specificity are essential for the appropriate triaging and treatment of incoming patients. The assay has a limit of detection of 250 copies/ml, which is in line with that claimed by other commercial SARS-CoV-2 tests (20, 21). The negative percent agreement of the SAMBA SARS-CoV-2 test in clinical samples was 96.4% and the positive percent agreement was 98.9% compared to the centralized molecular testing by CMPHL. These data include 3 positive samples detected by SAMBA that were originally negative by centralized testing, indicating good sensitivity. In a separate study, SAMBA II was shown to have a sensitivity of 96.9% (95% CI, 84.2 to 99.9%) and specificity of 100% (95% CI, 96.9 to 100%) in accident and emergency (A&E) settings compared to the centralized reference method (17). Clinical evaluation by Zhen et al. (22) comparing the performance of Xpert Xpress SARS-CoV-2 (Cepheid), ePlex SARS-CoV-2 (GenMark), and ID Now COVID-19 (Abbott) assays showed limits of detection of 100 copies/ml, 1,000 copies/ml, and 10,000 copies/ml, respectively, and clinical agreements with the reference standard of 98.3%, 91.4%, and 87.7%, respectively.

Potential limitations of the study are that clinical samples were collected in VTM and diluted 1:2 in SCoV buffer rather than collected directly into SCoV buffer, which may affect the sensitivity. For this study, testing was carried out by Diagnostics for the Real World personnel in laboratory settings and not at the POC.
